# Digital Technology Enablers of Tele-Neurorehabilitation in Pre- and Post-COVID-19 Pandemic Era – A Scoping Review

**DOI:** 10.5195/ijt.2024.6611

**Published:** 2024-06-28

**Authors:** Mohy Uddin, Krishnan Ganapathy, Shabbir Syed-Abdul

**Affiliations:** 1 Research Quality Management Section, King Abdullah International Medical Research Center, King Saud bin Abdulaziz University for Health Sciences, Ministry of National Guard Health Affairs, Riyadh, Saudi Arabia; 2 Distinguished Visiting Professor IIT Kanpur & Director Apollo Telemedicine Networking Foundation, India; 3 Graduate Institute of Biomedical Informatics, College of Medical Science and Technology, Taipei Medical University, Taipei, Taiwan

**Keywords:** COVID-19, Neurorehabilitation, Telehealth, Telemedicine, Tele-Neurorehabilitation

## Abstract

Neurorehabilitation (NR), a major component of neurosciences, is the process of restoring a patient's damaged/disorganized neurological function, through training, therapy, and education, while focusing on patient's independence and well-being. Since the advent of the COVID-19 pandemic, various applications of telecare and telehealth services surged drastically and became an integral part of current clinical practices. Tele-Neurorehabilitation (TNR) is one of such applications. When rehabilitation services were disrupted globally due to lockdown and travel restrictions, the importance of TNR was recognized, especially in developed, low, and middle-income countries. With exponential deployment of telehealth interventions in neurosciences, TNR has become a distinct stand-alone sub-specialty of neurosciences and telehealth. Digital technologies, such as wearables, robotics, and Virtual Reality (VR) have enabled TNR to improve the quality of patients' lives. Providing NR remotely using digital technologies and customized digital devices is now a reality, and likely to be the new norm soon. This article provides an overview of the needs, utilization, and deployment of TNR, and focuses on digital technology enablers of TNR in pre- and post-COVID-19 pandemic era.

Neurological disorders affect almost one billion people globally. The prevalence of disability due to neurological disorders is rising worldwide, creating a significant disability burden that will require comprehensive management, including rehabilitation (Feigin et al., 2020). According to a global estimate, one in three people worldwide will need rehabilitation services at some time, and an estimated 2.4 billion individuals have health conditions that may benefit from some form of rehabilitation (Cieza et al., 2020).

Neurorehabilitation (NR) is an inter-disciplinary and cross-sectional health service for people with disease or injury to the neurological system. NR aims to optimize health, promote well-being, and enhance persons' abilities to have a good quality of life (Cummins et al., 2022). NR is critical in enhancing functional recovery in neurological disorders, and helps maintain, restore, and improve skills lost or impaired due to neurological conditions. The conventional NR approach is based on the physical and occupational therapy sessions done mainly in one-to-one meetings with therapists during either inpatient or outpatient hospital settings, is quite resource demanding and faces many challenges, such as increasing number of patients, shortage of expert staff, and economic pressure of cost minimization (Lambercy et al., 2021).

With the resurgence of COVID-19 pandemic, lockdown, quarantine, and travel restrictions, the accessibility to hospitals, emergency care, and rehabilitation services was drastically reduced. The pandemic brought further constraints to the traditional NR approach, because in-person NR could increase risk of possible exposure to COVID-19 (Caso & Federico, 2020). The importance and need to utilize modern digital technology enabled telemedicine/telehealth interventions became very clear in that scenario. Due to COVID-19 restrictions, utilization of digital technologies to deliver NR was not a question of when, but rather a question of how and now! The advancements brought by the digital revolution addressed the limitations of the current healthcare model faced during the pandemic by providing high quality rehabilitation in patients' homes along a continuum of care (Galea, 2019).

Telerehabilitation (TR), an emerging and advanced approach to providing remote healthcare, is the delivery of rehabilitation services using telecommunication/digital technologies (Alexander, 2022). TR overcomes the barriers of distance and time by providing patients with disabilities access to accurate diagnosis and treatment. User-friendly digital technologies that are flexible, simple, intuitive, with a tolerance for error, requiring low physical effort, and controlled remotely, can do wonders. In TR, remotely located patient-centric devices are integrated with communication and software systems. These need to work seamlessly with the patients having impairments of fine/gross motor skills, cognition, speech, language, vision and hearing (Brennan et al., 2010). The examples of TR systems include: Virtual Reality (VR), video games, web-based interventions, mobile apps, and online/telephonic telecoach programs (Matamala-Gomez et al., 2020). These systems play a key role in facilitating the relationship between clinicians and patients, and enable the continuity of the rehabilitation process for patients in their homes. TR, which is delivered virtually, ensures the access to high-quality, cost-effective care despite isolation, reduces travel and waiting time, and improves quality of life and patients' outcomes.

Tele-Neurorehabilitation (TNR) is an innovative approach that allows neurologically afflicted patients, who might not otherwise have access to healthcare facilities due to distance or economic issues, to continue cost effective rehabilitation at home (Khanna et al., 2018). Numerous published studies (Brennan et al., 2021; Cerfoglio et al., 2023; Cox et al., 2021; Iodice et al., 2021; Maresca et al., 2020; Matamala-Gomez et al., 2021; Srivastava et al., 2021) demonstrated the efficacy of providing remote, home-based telerehabilitation solutions to patients with neurological diseases during the COVID-19 pandemic.

Modern digital technologies facilitate e-visits for remote patient assessment, monitoring, and care. Various methods and components of TNR using technological support have been described in the literature for variety of diseases (Ganapathy, 2021; Garg & Dhamija, 2020; Golomb et al., 2010; Klaic & Galea, 2020; Matamala-Gomez et al., 2020; Montana et al., 2020; Peretti et al., 2017; Rogante et al., 2010; Stasolla et al., 2023; Tressoldi et al., 2012). In a recent systematic review (Matamala-Gomez et al., 2020) that assessed the role and effectiveness of different engagement strategies in TNR for patients with neurological disorders, out of 18 analyzed studies, 12 studies reported positive results, five studies reported neutral effects, and only one study reported negative results. Examples of TNR services include: physical motor therapy, occupational therapy, speech-language therapy, audiology, VR, telerobotic therapy, and gamification. Virtual physical therapy includes assessment, intervention, monitoring, education, and training. Two-way real-time virtual visits are enabled using audio-visual tools and equipments. Examples include: video calls, video conferencing, asynchronous e-visits, virtual check-ins, and remote evaluations of recorded images and videos. Similarly, machines interfaced with smart devices like robotic arms, robotic legs, data gloves, and smart glasses in a 3D environment allow a greater sense of immersion in the virtual environment. Technology plays a major role in TNR. Deployment of technology should always be contextual, user friendly, flexible, simple, intuitive, and cost effective. Complex technology (i.e., multiple components, settings and/or connections) may be associated with higher cost and a steeper learning curve, leading to eventual non-use of the system. TNR enabled devices help a therapist monitor a patient remotely using applications installed on a tablet, smartphone, or customised smart gloves and splints. Patients can practice exercises independently even without a therapist being online. Fuzzy Inference Systems (FIS) have remote monitoring tools that assist therapists to supervise patients' exercises remotely.

## Search Strategy and Selection Criteria

A scoping review was performed to search the scientific and academic literature for related studies in the field of TNR focusing on the digital technology aspects and covering the time period of the pre- and post-COVID-19 pandemic era. For the search, we used the PubMed database and the Google Scholar search engine. The rationale for using both was that they are the most widely used, verified, and freely accessible resources for searching high quality scientific and academic publications online. For the search terms, we used Medical Subject Heading (MeSH) terms via the NIH MeSH Browser. The search was performed using various combinations of terms using Boolean operator ‘AND.’ The search terms included: ‘Neurorehabilitation,’ ‘Neurological Rehabilitation,’ ‘Digital Technology,’ ‘Telemedicine,’ ‘Telehealth,’ ‘Neurology,’ and ‘COVID-19'.

For the selection of studies, the following criteria were followed:

- Studies in the field of TNR
- Studies that focused on the Digital Technology aspects
- Studies covering the timespan of pre- and post-COVID pandemic era
- Studies written in the English language

For the exclusion of studies, the following criteria was followed:

- Non-peer reviewed literature
- Study types categorized as viewpoints, letters, and guidelines

All selected studies matching the inclusion and exclusion criteria were reviewed in detail. The data were extracted, summarized, and discussed for the main digital technologies involved in TNR in the pre- and post-COVID-19 pandemic era.

## Key Digital Technology Enablers of Tele-Neurorehabilitation

Digital technologies enable TNR to improve quality of life for patients particularly during pandemics, lockdowns, quarantines, and social distancing preventing scenarios. Digital technologies provide various platforms that can enable remote patient monitoring asynchronously. These asynchronous platforms include: wikis, blogs, forums, or e-mail. Communication can occur synchronously through instant messaging, videoconferencing, and chat servers (Mosca et al., 2020). Multimedia technologies enable interaction and sensory feedback through motivating multidimensional virtual environments. Video game consoles assist in motor rehabilitation (Gutiérrez et al., 2013).

Smart chatbots (e.g. Siri) and future-generation machine learning ensure intelligent engagement (Dobkin, 2016). USB-based wrist blood pressure cuffs, mats with contact sensitive switches, gaming driving wheels with special grippers, and joysticks are used for stroke patients. Socially assistive humanoid robots, which are safe, portable and user-friendly, deliver emotional support (Lambercy et al., 2021). There is a variety of technological approaches proposed as well as implemented for TNR, from simple mobile phone applications to advanced AI-based applications supported by passive instrumented tools (Nijenhuis et al., 2017). We examined some of the key digital technology enablers that played a pivotal role in providing quality TNR with focus on the pre- and post-COVID-19 pandemic era.

## Wearables

Wearable or wearable devices are the electronic devices that can be worn on the body or incorporated into clothing or body-worn accessories by individuals, and are used for tracking, measuring, and analysing different activities, parameters and indictors (Smuck et al., 2021). One of the primary uses of wearable devices is in the field of healthcare. They occur in various domains, such as biomedical research, clinical care, and personalized medicine (Canali et al., 2022). Wearable sensors are important for objective and continuous measurement of motor activities, such as lying, sitting, standing, and walking; and these devices are valuable in home care as well as in clinical research (Adams et al., 2017). Smart wearable mechatronic systems, that measure and display body signals, provide haptic, vibro, and electro feedback stimulation (Atashzar et al., 2021). A wearable device with multiple small accelerometers and gyroscope sensors captures and wirelessly transmits data to assess the health of the patients affected by movement disorders. These wearable accelerometers have been successfully used in various studies for the assessment of gait performance and long-term measurements of mobility in patients with multiple sclerosis (MS) in clinical settings, especially for those with mild to moderate disability in the past two decades (Motl et al., 2013; Neven et al., 2016; Ng & Kent-Braun, 1997; Pau et al., 2016; Pearson et al., 2004; Sosnoff et al., 2012). Unobtrusive wearable inertial measurement units (IMUs) have been used for the objective evaluation and long-term monitoring of motor symptoms in patients with Parkinson's disease (PD) (Suppa et al., 2017; Weiss et al., 2015). Due to these features, the clinical demand for use of wearables to objectively collect and quantitatively analyse the data has been on rise (Jeon et al., 2017; Maetzler et al., 2013). Linear and axial speeds and falls, gross and fine motor movements, gait, balance, bradykinesia, tremor, rigidity, and non-motor symptoms can be measured with these devices (Asakawa et al., 2019). Instrumented insoles are connected wirelessly to a 4G / 5G ready tablet, PC, a server, and a web-interface for experts. Movement parameters are delivered to a health platform (Jagos et al., 2015). Wearable wireless inertial sensors on ankles communicate with smartphones. Type, quantity and quality of domiciliary activities are collated using signal processing algorithms that can be reviewed on a weekly basis. Accelerometer based motion sensing technologies have been used. Xbox 360® and Kinect console have been used in management of MS patients. Remote step count monitoring using a consumer-friendly accelerometer (Fitbit Flex) enhances MS disability assessment. Lower average daily step count is associated with greater MS disability (Block et al., 2017). Custom-built Inertia Measurement sensors with video cameras estimating posture improve patients' coverage (Paloschi et al., 2021). Cloud database exergames on smart phones are deployed in TNR (Burgos et al., 2020). Tele-assessment systems acquire kinematic data of forward reaching movements in stroke patients. Correlation coefficients of reaching displacement, velocity and acceleration measurements are obtained (Rau et al., 2013). Wearable sensors allow quantitative monitoring of a patient's performance. Remote signal processing from wearables assists in the prediction of long-term dynamics of patient recovery. Brain computer interfaces (BCIs) have been extensively used for patients with a variety of neuromuscular disorders and their effectiveness in the recovery of neural functions has been confirmed, especially for stroke patients in different studies (Mansour et al., 2022; Padfield et al., 2022; Prasad et al., 2010). Similarly, several studies have demonstrated benefits of motor-imagery-based systems for patients having a variety of neurological diseases (Lee & Hwang, 2019; López-Larraz et al., 2015). Being safe and economical for home based therapy, motor imagery based BCIs provide a novel rehabilitation paradigm by substituting or supplementing current therapy protocols with the support of several training sessions (Arpaia et al., 2023).

## Robotics

In the recent past, robots have contributed substantially by providing novel tools to the clinician for patient treatment. AI enabled tele, autonomous, collaborative, hand-held, social and exoskeleton robots facilitate interaction between an in-home patient and in-clinic therapies. There are two general configurations of TR robotic systems: the first one is unilateral, and used most commonly, in which only the patient can interface with the robots; the second is bilateral wherein both patients and therapists can use the robots and interact with each other using a shared virtual environment. TNR assisted by robotics provides innovative, interactive, and reproducible therapies for a longer time and can be implemented site-to-site (Carignan & Krebs, 2006). Socially assistive robots provide psychological support and benefit elderly patients with autism, mild cognitive impairments, and dementia (Tulsulkar et al., 2021). Conventional robotic rehabilitation enables vibrotactile and haptic feedback. The wearable robotics (i.e., exoskeletons) have been used for various VR applications, and provide a natural haptic interface for patient interaction and a surround feel environment for the training (Carignan & Krebs, 2006).

Existing robotic rehabilitation systems are expensive, bulky, and have safety concerns (Atashzar et al., 2021). However, various studies have shown the efficacy of therapy robots in neurological rehabilitation thus proving the usefulness of these robots as an alternative system or an integrative one with classical methods (Hidler & Sainburg, 2011; Huang & Krakauer, 2009; Iandolo et al., 2019; Kuo et al., 2021; Ona et al., 2018; Semprini et al., 2018). These robots can be easily controlled from remote locations via different telecommunication systems that makes them suitable for home therapy and a prolonged rehabilitation process (Mobini et al., 2013). The transfer of the new technology from the rehabilitation hospital to a patient's home is a major challenge of current technological and clinical research (Johnson & Schmidt, 2009).

The use of robotics and other similar interactive devices has great opportunities to widen treatment options and improve outcomes of patients with neurological disabilities and impairments, and their advantages are proven through the clinical trials (Fazekas & Tavaszi, 2019). These advantages signify that the potential usefulness of robots can be further improved by upcoming technical developments within targeted rehabilitation settings. Long-term evaluation of social robotic platforms in TNR could confirm beneficial effects. Because these robots might pose hazards for patients, therefore safety considerations and precautions must be considered before their deployment.

## Virtual Reality

VR is another novel intervention of digital technology that can recreate realistic environments in which patients may bodily operate and improve balance. Depending on the intensity and quality of emotions elicited in the computer generated world (Mancuso et al., 2023), different types of VR can be differentiated including: Non-Immersive VR (Ventura et al., 2019), Fully Immersive VR (Bohil et al., 2011; Matthews, 2018; Sanchez-Vives & Slater, 2005), Augmented Reality (Porter & Heppelmann, 2017), Mixed Reality (Park et al., 2019), and Extended Reality (Georgiev et al., 2021). Incorporation of VR is one of the widely used methods for TR to make the treatment as safe as a conventional rehabilitation method. Some of the key applications of VR in NR include: motor rehabilitation, cognitive rehabilitation, emotional rehabilitation, and sensory rehabilitation (Georgiev et al., 2021). VR environments coupled with robotic systems, visual, haptic, and auditory cues can be fused with kinesthetic cues enabling multimodal goal-oriented sensorimotor tasks. Multisensory stimulation in a realistic environment motivates and ensures adherence and better clinical results (Burgos et al., 2020). The literature has numerous studies in which VR is effective in training balance and gait in patients with stroke, PD, MS, cerebral palsy and neurological or vestibular diseases (Feintuch et al., 2014; Garg & Dhamija, 2020; Goffredo et al., 2023; Maldonado-Díaz et al., 2021; Nieto-Escamez et al., 2023; Saladino et al., 2023; Truijen et al., 2022). Due to augmented feedback for the performance with various inputs, VR balance training gives an edge by stimulating both motor and cognitive processes as compared to traditional balance training (Li et al., 2016; Phu et al., 2019). VR therapy satisfies the criteria of an effective treatment method by providing cognitive motor training, motivational activities, and empowerment techniques consistent with evidence-based neuroscience principles. VR and robotic systems provide motivation by making exercises comfortable, safe, engaging and entertaining, hence boosting the self-confidence, self-management, self-efficacy, self-reliance, and improved quality of life for the patients (Maldonado-Díaz et al., 2021; Mumford et al., 2012; Perez-Marcos et al., 2018; Ustinova et al., 2014). Recent meta-analysis, bibliometric analysis, and review reports of VR in NR highlight the benefits of VR in neurological disorder management and improvements in motor function, fine motor skills, mobility, balance, and upper limb function, hence bringing improvements to the quality of life of patients (Georgiev et al., 2021; Guo et al., 2022; Massetti et al., 2018; Montana et al., 2020; Truijen et al., 2022; Voinescu et al., 2021). Clinicians can now immerse their patients in different virtual worlds to reduce pain and anxiety and encourage them to move by playing games. However, when developing VR systems that provide effective and safe interventions that improve patients' outcomes, there must be an understanding of the patients' characteristics and the clinical criteria of physiotherapy (Glegg et al., 2013; Landis & Koch, 1977). VR based TNR is an excellent approach for the continuity of patient treatment that can be used widely due to its support for clinical decision making in the rehabilitation process (Maldonado-Díaz et al., 2021). Acceptance of VR implies familiarity and confidence in the technology.

## Summary of Key Digital Technology Enablers of Tele-Neurorehabilitation

Table 1 below provides a brief summary of selected studies and shows their characteristics, i.e., type of devices that enable TNR, reasons for their use, and study population.

**Table 1:** Summary of Key Digital Technology Enablers of Tele-Neurorehabilitation

S. No	Author (Year of Study)	Title of Study	Digital Technology Enabler Devices (Wearables / VR / Robotics)	Reason of Devices Used	Study Population
1	(Gutiérrez et al., 2013)	A telerehabilitation program improves postural control in multiple sclerosis patients: A Spanish preliminary study	Video game consoles	Motor rehabilitation	People with MS
2	(Dobkin, 2016)	Behavioral self-management strategies for practice and exercise should be included in neurologic rehabilitation trials and care	Smart chatbots and future-generation machine learning enabled Siri	Intelligent engagement	People with disabilities
3	(Lambercy et al., 2021)	Neurorehabilitation from a distance: Can intelligent technology support decentralized access to quality therapy?	USB based wrist blood pressure cuff, mat with contact sensitive switches, gaming driving wheel with a special gripper, and joysticks	Video/audio support	People with stroke
			Socially assistive humanoid robots	Emotional support	Remote patients
4	(Nijenhuis et al., 2017)	Effects of training with a passive hand orthosis and games at home in chronic stroke: A pilot randomized controlled trial	Passive hand orthosis and games	Arm and hand function improvements	People with stroke
5	(Smuck et al., 2021)	The emerging clinical role of wearables: Factors for successful implementation in healthcare	Body-worn wearable devices and accessories	Tracking, measuring and analyzing different activities, parameters and indictors	All populations
6	(Adams et al., 2017)	Multiple wearable sensors in Parkinson and Huntington disease individuals: A pilot study in clinic and at home	Multiple wearables sensors	Objective and continuous measurement of motor features	People with PD or Huntington disease
7	(Atashzar et al., 2021)	Review: How can intelligent robots and smart mechatronic modules facilitate remote assessment, assistance, and rehabilitation for isolated adults with neuro-musculoskeletal conditions?	Smart wearable mechatronic systems	Measuring and displaying body signals	Adults with neuro-musculoskeletal conditions
8	(Motl et al., 2013; Neven et al., 2016; Ng & Kent-Braun, 1997; Pau et al., 2016; Pearson et al., 2004; Sosnoff et al., 2012)	Accelerometry as a measure of walking behavior in multiple sclerosis Understanding walking activity in multiple sclerosis: Step count, walking intensity and uninterrupted walking activity duration related to degree of disability Quantification of lower physical activity in persons with multiple sclerosis Clinical assessment of gait in individuals with multiple sclerosis using wearable inertial sensors: Comparison with patient-based measure Quantification of walking mobility in neurological disorders Falls and physical activity in persons with multiple sclerosis	Wearable devices, accelerometers, and gyroscope sensors	Assessment of gait performance and long-term measurements of mobility	People with movement disorders, neurological disorders, or MS
9	(Suppa et al., 2017; Weiss et al., 2015)	L-dopa and freezing of gait in Parkinson's disease: Objective assessment through a wearable wireless system New evidence for gait abnormalities among Parkinson's disease patients who suffer from freezing of gait: insights using a body-fixed sensor worn for 3 days	Wearable inertial measurement units (IMUs)	Objective evaluation and long-term monitoring of motor symptoms	People with PD
10	(Jeon et al., 2017; Maetzler et al., 2013) (Asakawa et al., 2019)	Automatic classification of tremor severity in Parkinson's disease using a wearable device Quantitative wearable sensors for objective assessment of Parkinson's disease Can the latest computerized technologies revolutionize conventional assessment tools and therapies for a neurological disease? The example of Parkinson's disease	Wearable devices and wearable sensors	Objective collection and quantitative analysis of the data; measurements of linear and axial speeds and falls, and gross and fine motor movements, and non-motor symptoms	People with PD
11	(Jagos et al., 2015)	A framework for (tele-) monitoring of the rehabilitation progress in stroke patients	Instrumented insoles	Delivery of movement parameters	People with stroke
12	(Block et al., 2017)	Continuous daily assessment of multiple sclerosis disability using remote step count monitoring	Wireless inertial sensors, Xbox 360® and Kinect consoles, and Fitbit Flex	Accelerometry based motion sensing, remote step count monitoring, and disability assessment	People with MS
13	(Paloschi et al., 2021)	Validation and assessment of a posture measurement system with magneto-inertial measurement units	Inertia Measurement sensors	Improving patients' coverage	People with lower back pain (LBP)
14	(Burgos et al., 2020)	Exergames and telerehabilitation on smartphones to improve balance in stroke patients	Exergames and sensors	Estimating posture, improving patients' coverage, adherence, and clinical results	People with stroke
15	(Mansour et al., 2022; Padfield et al., 2022; Prasad et al., 2010)	Efficacy of brain–computer interface and the impact of its design characteristics on poststroke upper-limb rehabilitation: A systematic review and meta-analysis of randomized controlled trials A comprehensive review of endogenous EEG-based BCIs for dynamic device control Applying a brain-computer interface to support motor imagery practice in people with stroke for upper limb recovery: A feasibility study	Brain computer interfaces (BCIs)	Recovery of neural functions	People with a variety of neuromuscular disorders, and people with stroke
16	(Lee & Hwang, 2019; López-Larraz et al., 2015)	Motor imagery on upper extremity function for persons with stroke: A systematic review and meta-analysis Evolution of EEG motor rhythms after spinal cord injury: A longitudinal study	Motor-imagery-based BCIs / systems	Screening, selecting and monitoring patients in rehabilitation	People having a variety of neurological diseases and people with stroke
17	(Tulsulkar et al., 2021)	Can a humanoid social robot stimulate the interactivity of cognitively impaired elderly? A thorough study based on computer vision methods	Socially assistive robots	Providing psychosocial support and interventions	Older adults with cognitive impairments
18	(Carignan & Krebs, 2006)	Telerehabilitation robotics: bright lights, big future?	Wearable robotics (exoskeletons)	Providing natural haptic interface for patient interaction and surround feel environment for the training	Older adults
19	(Hidler & Sainburg, 2011; Huang & Krakauer, 2009; Iandolo et al., 2019; Kuo et al., 2021; Ona et al., 2018; Semprini et al., 2018).	Role of Robotics in Neurorehabilitation Robotic neurorehabilitation: A computational motor learning perspective Perspectives and challenges in robotic neurorehabilitation Prediction of robotic neurorehabilitation functional ambulatory outcome in patients with neurological disorders A review of robotics in neurorehabilitation: Towards an automated process for upper limb Technological approaches for neurorehabilitation: From robotic devices to brain stimulation and beyond	Therapy robots	Neurological rehabilitation, home therapy, and prolonged rehabilitation process	People with neurological disorders, disabilities, and impairments
20	(Feintuch et al., 2014; Garg & Dhamija, 2020; Goffredo et al., 2023; Maldonado-Díaz et al., 2021; Nieto-Escamez et al., 2023; Saladino et al., 2023; Truijen et al., 2022)	Virtual reality applications in neurorehabilitation Teleneurorehabilitation for Parkinson's disease: A panacea for the times to come? Non-immersive virtual reality telerehabilitation system improves postural balance in people with chronic neurological diseases Teleneurorehabilitation program (virtual reality) for patients with balance disorders: A descriptive study Virtual reality applications in neurorehabilitation: Current panorama and challenges Neuro rehabilitation effectiveness based on virtual reality and tele rehabilitation in people with multiple sclerosis in Argentina: Reavitelem study Effect of home-based virtual reality training and telerehabilitation on balance in individuals with Parkinson disease, multiple sclerosis, and stroke: A systematic review and meta-analysis	Immersive, semi-immersive, or non-immersive VR Systems / devices, personalized VR systems, bespoke VR systems, and commercial VR devices	Training balance and gait, improving posture balance and posture control, improving functional mobility, management of acute and chronic pain, and promoting well-being	People with stroke, PD, MS, cerebral palsy, dementia, and patients with neurological or vestibular diseases or balance disorders, and older adults
21	(Li et al., 2016; Phu et al., 2019)	Virtual reality for improving balance in patients after stroke: A systematic review and meta-analysis Balance training using virtual reality improves balance and physical performance in older adults at high risk of falls	WiiFit, Balance Rehabilitation Unit (BRU), and other computer-based systems	Falls prevention, balance control, and improving balance	People with stroke and older adults
22	(Maldonado-Díaz et al., 2021; Mumford et al., 2012; Perez-Marcos et al., 2018; Ustinova et al., 2014)	Teleneurorehabilitation program (virtual reality) for patients with balance disorders: Descriptive study Upper-limb virtual rehabilitation for traumatic brain injury: A preliminary within-group evaluation of the elements system Virtual reality as a vehicle to empower motor-cognitive neurorehabilitation Virtual reality game-based therapy for treatment of postural and co-ordination abnormalities secondary to TBI: A pilot study	VR and robotic systems	Empowering motor-cognitive NR, boosting the self-confidence, self-management, self-efficacy, and self-reliance	People with balance disorders and brain injury
23	(Georgiev et al., 2021; Guo et al., 2022; Massetti et al., 2018; Montana et al., 2020; Truijen et al., 2022; Voinescu et al., 2021).	Virtual reality for neurorehabilitation and cognitive enhancement Virtual reality for neurorehabilitation: A bibliometric analysis of knowledge structure and theme trends The clinical utility of virtual reality in neurorehabilitation: A systematic review The benefits of emotion regulation interventions in virtual reality for the improvement of wellbeing in adults and older adults: A systematic review Effect of home-based virtual reality training and telerehabilitation on balance in individuals with Parkinson's disease, multiple sclerosis, and stroke: A systematic review and meta-analysis Virtual reality in neurorehabilitation: An umbrella review of meta-analyses	Interactive VR devices combined with computer assisted systems, VR-based treadmill, virtual objects, game platforms (such as Nintendo WII or Xbox Kinect etc.), interaction devices, sensory display systems, virtual environments, and custom-designed devices	Motor rehabilitation, cognitive rehabilitation, emotional rehabilitation, sensory rehabilitation, and self-enhancement	People with traumatic brain injury (TBI), stroke, neurological disorders, tumor, and chronic conditions; and older adults

## Challenges in Implementing Tele-Neurorehabilitation

Aside from the above strengths and benefits, TNR has various social and technical challenges along with the limitations (Ganapathy, 2021). Many TNR needs cannot be met in low resource settings. Access to smart phones and computers may not be universal. There could be difficulty in using devices and softwares effectively. Power, network, bandwidth issues, shortage of multimedia devices, licensing, liability, malpractice, affordability, reduced motivation, considering virtual consults as ‘impersonal', and lack of physical space at home are some of the practical challenges in implementing TNR. Usually, persons who are elderly require repeated instructions for TNR. Poor lighting, hearing impairments, call drop, difficulty in remembering procedures, lags during conversation, and difficulty in troubleshooting are additional concerns. The therapist often needs to see the whole person on the screen, which may not be possible due to the limitations of tele-services. On the other hand, in-person services have several advantages, including socialization, building self-esteem, learning instrumental activities, mentorship, and hands-on training. Overall, there are multiple barriers associated with TNR practices, in which privacy, confidentiality, and safety are the main concerns (Khanna et al., 2018). Similarly, when dealing with advanced technological procedures, the acceptance of technology and the patients' confidence in technology, especially in persons who are elderly, is another important factor that needs attention. Preliminary training of both patients and care providers is required to implement advanced tele-rehabilitative procedures, and continuous and supervised monitoring is required during the exercises for the critically ill patients or patients with other comorbidities (Nuara et al., 2022). Not much research is done for the policy and license issues along with the cross-cultural acceptance of TNR services (McCue et al., 2010). Proper governance to strengthen the health care systems thorough surveillance, logistics, and service delivery is required. Strong IT infrastructure and compatible technologies are pivotal for sustainable and effective TR services. Ultimately, incorporating telemedicine infrastructures like TNR in the patient care domain ensures a pre-emptive, viable and robust approach that can withstand future global pandemics, if needed (Calabro, 2021).

## Conclusions

The results from this article show that, with the latest trends and advancements in digital technology, TNR is becoming a part of the new norm for the long-term telecare practices. In the future, TNR will eventually be integrated with Smart Homes in Smart Cities. Functional monitoring with bed sensors, activity, motion sensors, and gait monitors will be possible. Creating a connected home with pressure-sensing floors, smart furniture, medical sensors, and integrating smart appliances needs to be contextual. Assistive robots, smart wheelchairs, prosthetic limb controls, home automation systems, and an AI Chatbot companion at home will add value, providing 'smarter care'. The latter would include medication reminders, contacting caregiver/children in emergencies, encouraging activities, and making appointments. Eventually, staying at home or aging in place will likely lead to better health outcomes.

The COVID-19 pandemic has resulted in an exponential growth of TNR. Studies utilizing TNR for persons with stroke, PD, neurotrauma, and neurodegenerative disorders have been reported in the above-mentioned literature. Use of modern digital technologies that enable TNR will become common. Identifying specific use cases will help in achieving customized, well defined, and changing goals. Ultimately, what really matters is the patient's goals; TNR is only a service to help achieve them. One needs to 'get into the mind' of the end user. 'Customer delight' is not a cliché used as a marketing ploy. TNR should not be a solution in search of a problem. In the evolving patient-centric approach, the patients' role is changing. The ‘digitally engaged’ patient is now at the centre of self-care. Technology acceptance, behavioural modification, increasing digital infrastructure, and patients' requirements are the key components that will trigger the paradigm shift in the delivery of TNR services in the future. Beyond the COVID-19 pandemic, due to the social distance constraints experienced by immunocompromised patients and distancing limits for infectious disease patients, there is a clear need to rethink current NR routines. The optimum solution seems to be a hybrid model of delivering with both in-person sessions along with TNR.

Achieving technical success is different from demonstrating a clinical difference in health care outcomes. Evidence-based, thorough, and systematic evaluations are required for TNR. Like other disruptive technologies, the potential of TNR to cause a profound impact should not be underestimated. Technology deployment for clinical NR presupposes a thorough understanding of the technology - who will use it, when, where, why, and how it will help achieve pre-set goals.

## Declaration

**Data availability statement:** Data sharing is not applicable to this article as no datasets were generated or analyzed during the current study.

**Consent for publication:** Not applicable. This article does not contain any individual person's data.

**Ethical Approval:** Not applicable. This article does not contain any studies related to human or animal subjects.

**Funding:** The authors received no financial support for the research, authorship, and/or publication of this article.

## References

[R1] Adams, J. L., Dinesh, K., Xiong, M., Tarolli, C. G., Sharma, S., Sheth, N., Aranyosi, A. J., Zhu, W., Goldenthal, S., Biglan, K. M., Dorsey, E. R., & Sharma, G. (2017). Multiple wearable sensors in Parkinson and Huntington disease individuals: A pilot study in clinic and at home. Digital Biomarkers, 1(1), 52–63. 10.1159/000479018 32095745 PMC7015372

[R2] Alexander, M. (2022). Chapter 1 - Introduction. In M. Alexander (Ed.), Telerehabilitation (pp. 1-3). Elsevier. 10.1016/B978-0-323-82486-6.00001-0

[R3] Arpaia, P., Coyle, D., Esposito, A., Natalizio, A., Parvis, M., Pesola, M., & Vallefuoco, E. (2023). Paving the way for motor imagery-based tele-rehabilitation through a fully wearable BCI system. Sensors, 23(13), 5836. 10.3390/s23135836 37447686 PMC10346666

[R4] Asakawa, T., Sugiyama, K., Nozaki, T., Sameshima, T., Kobayashi, S., Wang, L., Hong, Z., Chen, S., Li, C., & Namba, H. (2019). Can the latest computerized technologies revolutionize conventional assessment tools and therapies for a neurological disease? The example of Parkinson's disease. Neurologia Medico-Chirurgica, 59(3), 69–78. 10.2176/nmc.ra.2018-0045 30760657 PMC6434424

[R5] Atashzar, S. F., Carriere, J., & Tavakoli, M. (2021). How can intelligent robots and smart mechatronic modules facilitate remote assessment, assistance, and rehabilitation for isolated adults with neuro-musculoskeletal conditions? Frontiers in Robotics and AI, 8(48). 10.3389/frobt.2021.610529 PMC8072151 33912593

[R6] Block, V. J., Lizée, A., Crabtree-Hartman, E., Bevan, C. J., Graves, J. S., Bove, R., Green, A. J., Nourbakhsh, B., Tremblay, M., Gourraud, P. A., Ng, M. Y., Pletcher, M. J., Olgin, J. E., Marcus, G. M., Allen, D. D., Cree, B. A., & Gelfand, J. M. (2017). Continuous daily assessment of multiple sclerosis disability using remote step count monitoring. Journal of Neurology, 264(2), 316–326. 10.1007/s00415-016-8334-6 27896433 PMC5292081

[R7] Bohil, C. J., Alicea, B., & Biocca, F. A. (2011). Virtual reality in neuroscience research and therapy. Nature Reviews Neuroscience, 12(12), 752–762. 10.1038/nrn3122 22048061

[R8] Brennan, D., Tindall, L., Theodoros, D., Brown, J., Campbell, M., Christiana, D., Smith, D., Cason, J., & Lee, A. (2010). A blueprint for telerehabilitation guidelines. International Journal of Telerehabilitation, 2(2), 31–34. 10.5195/ijt.2010.6063 25945175 PMC4296793

[R9] Brennan, K., Curran, J., Barlow, A., & Jayaraman, A. (2021). Telerehabilitation in neurorehabilitation: Has it passed the COVID test? Expert Review of Neurotherapeutics, 21(8), 833–836. 10.1080/14737175.2021.1958676 34282965

[R10] Burgos, P. I., Lara, O., Lavado, A., Rojas-Sepúlveda, I., Delgado, C., Bravo, E., Kamisato, C., Torres, J., Castañeda, V., & Cerda, M. (2020). Exergames and telerehabilitation on smartphones to improve balance in stroke patients. Brain Sciences, 10(11). 10.3390/brainsci10110773 PMC7690853 33114245

[R11] Calabro, R. S. (2021). Teleneurorehabilitation in the COVID-19 era: What are we doing now and what will we do next? Medical Sciences, 9(1), 15. 10.3390/medsci9010015 33668321 PMC8006037

[R12] Canali, S., Schiaffonati, V., & Aliverti, A. (2022). Challenges and recommendations for wearable devices in digital health: Data quality, interoperability, health equity, fairness. PLOS Digital Health, 1(10), e0000104. 10.1371/journal.pdig.0000104 36812619 PMC9931360

[R13] Carignan, C. R., & Krebs, H. I. (2006). Telerehabilitation robotics: Bdright lights, big future? Journal of Rehabilitation Research & Development, 43(5), 695–710. 10.1682/jrrd.2005.05.0085 17123209

[R14] Caso, V., & Federico, A. (2020). No lockdown for neurological diseases during COVID-19 pandemic infection. Neurological Sciences, 41(5), 999–1001. 10.1007/s10072-020-04389-3 32270358 PMC7138901

[R15] Cerfoglio, S., Capodaglio, P., Rossi, P., Verme, F., Boldini, G., Cvetkova, V., Ruggeri, G., Galli, M., & Cimolin, V. (2023). Tele-rehabilitation interventions for motor symptoms in COVID-19 patients: A narrative review. Bioengineering, 10(6), 650. 10.3390/bioengineering10060650 37370581 PMC10295123

[R16] Cieza, A., Causey, K., Kamenov, K., Hanson, S. W., Chatterji, S., & Vos, T. (2020). Global estimates of the need for rehabilitation based on the global burden of disease study 2019: A systematic analysis for the global burden of disease study 2019. The Lancet, 396(10267), 2006–2017. 10.1016/S0140-6736(20)32340-0 PMC7811204 33275908

[R17] Cox, N. S., Scrivener, K., Holland, A. E., Jolliffe, L., Wighton, A., Nelson, S., McCredie, L., & Lannin, N. A. (2021). A brief intervention to support implementation of telerehabilitation by community rehabilitation services during COVID-19: A feasibility study. Archives of Physical Medicine and Rehabilitation, 102(4), 789–795. 10.1016/j.apmr.2020.12.007 33417964 PMC7981192

[R18] Cummins, C., Payne, D., & Kayes, N. M. (2022). Governing neurorehabilitation. Disability and Rehabilitation, 44(17), 4921–4928. 10.1080/09638288.2021.1918771 33989096

[R19] Dobkin, B. H. (2016). Behavioral self-management strategies for practice and exercise should be included in neurologic rehabilitation trials and care. Current Opinion in Neurology, 29(6), 693–699. 10.1097/wco.0000000000000380 27608301 PMC5842701

[R20] Fazekas, G., & Tavaszi, I. (2019). The future role of robots in neuro-rehabilitation. Expert Review of Neurotherapeutics, 19(6), 471–473. 10.1080/14737175.2019.1617700 31090484

[R21] Feigin, V. L., Vos, T., Nichols, E., Owolabi, M. O., Carroll, W. M., Dichgans, M., Deuschl, G., Parmar, P., Brainin, M., & Murray, C. (2020). The global burden of neurological disorders: Translating evidence into policy. The Lancet Neurology, 19(3), 255–265. 10.1016/s1474-4422(19)30411-9 31813850 PMC9945815

[R22] Feintuch, U., Katz, N., Kizony, R., Rand, D., & Weiss, P. L. (2014). Virtual reality applications in neurorehabilitation. In S. Clarke, L. G. Cohen, G. Kwakkel, R. H. Miller, & M. E. Selzer (Eds.), Textbook of Neural Repair and Rehabilitation: Volume 2: Medical Neurorehabilitation (2 ed., Vol. 2, pp. 198-218). Cambridge University Press. 10.1017/CBO9780511995590.021

[R23] Galea, M. D. (2019). Telemedicine in rehabilitation. Physical Medicine and Rehabilitation Clinics, 30(2), 473–483. 10.1016/j.pmr.2018.12.002 30954160

[R24] Ganapathy, K. (2021). Tele-rehabilitation - The time has come. Asian Hospital & Healthcare Management, (53). https://www.asianhhm.com/information-technology/tele-rehabilitation

[R25] Garg, D., & Dhamija, R. K. (2020). Teleneurorehabilitation for Parkinson's disease: A panacea for the times to come? Annals of Indian Academy of Neurology, 23(5), 592–597. 10.4103/aian.AIAN_566_20 33623256 PMC7887501

[R26] Georgiev, D. D., Georgieva, I., Gong, Z., Nanjappan, V., & Georgiev, G. V. (2021). Virtual reality for neurorehabilitation and cognitive enhancement. Brain Sciences, 11(2). 10.3390/brainsci11020221 PMC7918687 33670277

[R27] Glegg, S. M., Holsti, L., Velikonja, D., Ansley, B., Brum, C., & Sartor, D. (2013). Factors influencing therapists' adoption of virtual reality for brain injury rehabilitation. Cyberpsychology, Behavior and Social Networking, 16(5), 385–401. 10.1089/cyber.2013.1506 23713844

[R28] Goffredo, M., Pagliari, C., Turolla, A., Tassorelli, C., Di Tella, S., Federico, S., Pournajaf, S., Jonsdottir, J., De Icco, R., Pellicciari, L., Calabrò, R. S., Baglio, F., & Franceschini, M. (2023). Non-immersive virtual reality telerehabilitation system improves postural balance in people with chronic neurological diseases. Journal of Clinical Medicine, 12(9), 3178. 10.3390/jcm12093178 37176618 PMC10179507

[R29] Golomb, M. R., McDonald, B. C., Warden, S. J., Yonkman, J., Saykin, A. J., Shirley, B., Huber, M., Rabin, B., AbdelBaky, M., & Nwosu, M. E. (2010). In-home virtual reality videogame telerehabilitation in adolescents with hemiplegic cerebral palsy. Archives of Physical Medicine and Rehabilitation, 91(1), 1–8. 10.1016/j.apmr.2009.08.153 20103390

[R30] Guo, Q.-F., He, L., Su, W., Tan, H.-X., Han, L.-Y., Gui, C.-F., Chen, Y., Jiang, H.-H., & Gao, Q. (2022). Virtual reality for neurorehabilitation: A bibliometric analysis of knowledge structure and theme trends. Frontiers in Public Health, 10. 10.3389/fpubh.2022.1042618 PMC9684719 36438265

[R31] Gutiérrez, R. O., Galán Del Río, F., Cano de la Cuerda, R., Alguacil Diego, I. M., González, R. A., & Page, J. C. (2013). A telerehabilitation program by virtual reality-video games improves balance and postural control in multiple sclerosis patients. NeuroRehabilitation, 33(4), 545–554. 10.3233/nre-130995 24029009

[R32] Hidler, J., & Sainburg, R. (2011). Role of robotics in neurorehabilitation. Topics in Spinal Cord Injury Rehabilitation, 17(1), 42–49. 10.1310/sci1701-42 21857778 PMC3157701

[R33] Huang, V. S., & Krakauer, J. W. (2009). Robotic neurorehabilitation: A computational motor learning perspective. Journal of NeuroEngineering and Rehabilitation, 6(1), 5. 10.1186/1743-0003-6-5 19243614 PMC2653497

[R34] Iandolo, R., Marini, F., Semprini, M., Laffranchi, M., Mugnosso, M., Cherif, A., De Michieli, L., Chiappalone, M., & Zenzeri, J. (2019). Perspectives and challenges in robotic neurorehabilitation. Applied Sciences, 9(15), 3183. 10.3390/app9153183

[R35] Iodice, F., Romoli, M., Giometto, B., Clerico, M., Tedeschi, G., Bonavita, S., Leocani, L., Lavorgna, L., Digital Technologies, W., & Social Media Study Group of the Italian Society of, N. (2021). Stroke and digital technology: A wake-up call from COVID-19 pandemic. Neurological Sciences, 42(3), 805–809. 10.1007/s10072-020-04993-3 33433756 PMC7801773

[R36] Jagos, H., David, V., Haller, M., Kotzian, S., Hofmann, M., Schlossarek, S., Eichholzer, K., Winkler, M., Frohner, M., Reichel, M., Mayr, W., & Rafolt, D. (2015). A framework for (tele-) monitoring of the rehabilitation progress in stroke patients: Ehealth 2015 special issue. Applied Clinical Informatics Journal, 6(4), 757–768. 10.4338/aci-2015-03-ra-0034 PMC4704043 26767068

[R37] Jeon, H., Lee, W., Park, H., Lee, H. J., Kim, S. K., Kim, H. B., Jeon, B., & Park, K. S. (2017). Automatic classification of tremor severity in Parkinson's disease using a wearable device. Sensors, 17(9). 10.3390/s17092067 PMC5621347 28891942

[R38] Johnson, M. J., & Schmidt, H. (2009). Robot assisted neurological rehabilitation at home: Motivational aspects and concepts for tele-rehabilitation. Public Health Forum, 17(4), 8. 10.1016/j.phf.2009.09.005

[R39] Khanna, M., Gowda, G. S., Bagevadi, V. I., Gupta, A., Kulkarni, K., RP, S. S., Basavaraju, V., Ramesh, M. B., Sashidhara, H. N., Manjunatha, N., Channaveerachari, N. K., & Math, S. B. (2018). Feasibility and utility of tele-neurorehabilitation service in India: Experience from a quaternary center. Journal of Neurosciences in Rural Practice, 9(4), 541–544. 10.4103/jnrp.jnrp_104_18 30271047 PMC6126315

[R40] Klaic, M., & Galea, M. P. (2020). Using the technology acceptance model to identify factors that predict likelihood to adopt tele-neurorehabilitation. Frontiers in Neurology, 11. 10.3389/fneur.2020.580832 PMC7738474 33343488

[R41] Kuo, C.-Y., Liu, C.-W., Lai, C.-H., Kang, J.-H., Tseng, S.-H., & Su, E. C.-Y. (2021). Prediction of robotic neurorehabilitation functional ambulatory outcome in patients with neurological disorders. Journal of NeuroEngineering and Rehabilitation, 18(1), 174. 10.1186/s12984-021-00965-6 34922571 PMC8684617

[R42] Lambercy, O., Lehner, R., Chua, K., Wee, S. K., Rajeswaran, D. K., Kuah, C. W. K., Ang, W. T., Liang, P., Campolo, D., Hussain, A., Aguirre-Ollinger, G., Guan, C., Kanzler, C. M., Wenderoth, N., & Gassert, R. (2021). Neurorehabilitation from a distance: Can intelligent technology support decentralized access to quality therapy? Frontiers in Robotics and AI, 8(126). 10.3389/frobt.2021.612415 PMC8132098 34026855

[R43] Landis, J. R., & Koch, G. G. (1977). The measurement of observer agreement for categorical data. Biometrics, 33(1), 159–174. 10.2307/2529310 843571

[R44] Lee, D., & Hwang, S. (2019). Motor imagery on upper extremity function for persons with stroke: A systematic review and meta-analysis. Physical Therapy Rehabilitation Science, 8(1), 52–59. 10.14474/ptrs.2019.8.1.52

[R45] Li, Z., Han, X.-G., Sheng, J., & Ma, S.-J. (2016). Virtual reality for improving balance in patients after stroke: A systematic review and meta-analysis. Clinical Rehabilitation, 30(5), 432–440. 10.1177/0269215515593611 26141808

[R46] López-Larraz, E., Montesano, L., Gil-Agudo, Á., Minguez, J., & Oliviero, A. (2015). Evolution of EEG motor rhythms after spinal cord injury: A longitudinal study. PLOS One, 10(7), e0131759. 10.1371/journal.pone.0131759 26177457 PMC4503564

[R47] Maetzler, W., Domingos, J., Srulijes, K., Ferreira, J. J., & Bloem, B. R. (2013). Quantitative wearable sensors for objective assessment of Parkinson's disease. Movement Disorders, 28(12), 1628–1637. 10.1002/mds.25628 24030855

[R48] Maldonado-Díaz, M., Vargas, P., Vasquez, R., Gonzalez-Seguel, F., Rivero, B., Hidalgo-Cabalín, V., & Gutierrez-Panchana, T. (2021). Teleneurorehabilitation program (virtual reality) for patients with balance disorders: Descriptive study. BMC Sports Science, Medicine and Rehabilitation, 13(1), 83. 10.1186/s13102-021-00314-z PMC8330090 34340687

[R49] Mancuso, V., Bruni, F., Stramba-Badiale, C., Riva, G., Cipresso, P., & Pedroli, E. (2023). How do emotions elicited in virtual reality affect our memory? A systematic review. Computers in Human Behavior, 146, 107812. 10.1016/j.chb.2023.107812

[R50] Mansour, S., Ang, K. K., Nair, K. P., Phua, K. S., & Arvaneh, M. (2022). Efficacy of brain–computer interface and the impact of its design characteristics on poststroke upper-limb rehabilitation: A systematic review and meta-analysis of randomized controlled trials. Clinical EEG and Neuroscience, 53(1), 79–90. 10.1177/15500594211009065 33913351 PMC8619716

[R51] Maresca, G., Maggio, M. G., De Luca, R., Manuli, A., Tonin, P., Pignolo, L., & Calabrò, R. S. (2020). Tele-neuro-rehabilitation in Italy: State of the art and future perspectives. Frontiers in Neurology, 11, 563375. 10.3389/fneur.2020.563375 33101176 PMC7554582

[R52] Massetti, T., da Silva, T. D., Crocetta, T. B., Guarnieri, R., de Freitas, B. L., Bianchi Lopes, P., Watson, S., Tonks, J., & de Mello Monteiro, C. B. (2018). The clinical utility of virtual reality in neurorehabilitation: A systematic review. Journal of Central Nervous System Disease, 10, 1179573518813541. 10.1177/1179573518813541 30515028 PMC6262495

[R53] Matamala-Gomez, M., Bottiroli, S., Realdon, O., Riva, G., Galvagni, L., Platz, T., Sandrini, G., De Icco, R., & Tassorelli, C. (2021). Telemedicine and virtual reality at time of COVID-19 pandemic: An overview for future perspectives in neurorehabilitation. Frontiers in Neurology, 12, 646902. 10.3389/fneur.2021.646902 33841313 PMC8027250

[R54] Matamala-Gomez, M., Maisto, M., Montana, J. I., Mavrodiev, P. A., Baglio, F., Rossetto, F., Mantovani, F., Riva, G., & Realdon, O. (2020). The role of engagement in teleneurorehabilitation: A systematic review. Frontiers in Neurology, 11, 354. 10.3389/fneur.2020.00354 32435227 PMC7218051

[R55] Matthews, D. (2018). Virtual-reality applications give science a new dimension. Nature, 557(7703), 127–128. 10.1038/d41586-018-04997-2 29713071

[R56] McCue, M., Fairman, A., & Pramuka, M. (2010). Enhancing quality of life through telerehabilitation. Physical Medicine and Rehabilitation Clinics of North America, 21(1), 195–205. 10.1016/j.pmr.2009.07.005 19951786

[R57] Mobini, A., Behzadipour, S., & Foumani, M. S. (2013). Robotics and tele-rehabilitation: Recent advancements, future trends. International Journal of Reliable and Quality E-Healthcare, 2(4), 1–13. 10.4018/ijrqeh.2013100101

[R58] Montana, J. I., Matamala-Gomez, M., Maisto, M., Mavrodiev, P. A., Cavalera, C. M., Diana, B., Mantovani, F., & Realdon, O. (2020). The benefits of emotion regulation interventions in virtual reality for the improvement of wellbeing in adults and older adults: A systematic review. Journal of Clinical Medicine, 9(2), E500. 10.3390/jcm9020500 PMC7073752 32059514

[R59] Mosca, I. E., Salvadori, E., Gerli, F., Fabbri, L., Pancani, S., Lucidi, G., Lombardi, G., Bocchi, L., Pazzi, S., Baglio, F., Vannetti, F., Sorbi, S., & Macchi, C. (2020). Analysis of feasibility, adherence, and appreciation of a newly developed tele-rehabilitation program for people with MCI and VCI. Frontiers in Neurology, 11, 583368–583368. 10.3389/fneur.2020.583368 33329326 PMC7728852

[R60] Motl, R., Pilutti, L., Sandroff, B., Dlugonski, D., Sosnoff, J., & Pula, J. (2013). Accelerometry as a measure of walking behavior in multiple sclerosis. Acta Neurologica Scandinavica, 127(6), 384–390. 10.1111/ane.12036 23240822

[R61] Mumford, N., Duckworth, J., Thomas, P. R., Shum, D., Williams, G., & Wilson, P. H. (2012). Upper-limb virtual rehabilitation for traumatic brain injury: A preliminary within-group evaluation of the elements system. Brain Injury, 26(2), 166–176. 10.3109/02699052.2011.648706 22360522

[R62] Neven, A., Vanderstraeten, A., Janssens, D., Wets, G., & Feys, P. (2016). Understanding walking activity in multiple sclerosis: Step count, walking intensity and uninterrupted walking activity duration related to degree of disability. Neurological Sciences, 37, 1483–1490. 10.1007/s10072-016-2609-7 27207680

[R63] Ng, A. V., & Kent-Braun, J. A. (1997). Quantitation of lower physical activity in persons with multiple sclerosis. Medicine and Science in Sports and Exercise, 29(4), 517–523. 10.1097/00005768-199704000-00014 9107635

[R64] Nieto-Escamez, F., Cortés-Pérez, I., Obrero-Gaitán, E., & Fusco, A. (2023). Virtual reality applications in neurorehabilitation: Current panorama and challenges. Brain Sciences, 13(5). 10.3390/brainsci13050819 PMC10216098 37239291

[R65] Nijenhuis, S. M., Prange-Lasonder, G. B., Stienen, A. H., Rietman, J. S., & Buurke, J. H. (2017). Effects of training with a passive hand orthosis and games at home in chronic stroke: A pilot randomised controlled trial. Clinical Rehabilitation, 31(2), 207–216. 10.1177/0269215516629722 26869596

[R66] Nuara, A., Fabbri-Destro, M., Scalona, E., Lenzi, S. E., Rizzolatti, G., & Avanzini, P. (2022). Telerehabilitation in response to constrained physical distance: An opportunity to rethink neurorehabilitative routines. Journal of Neurology, 269(2), 627–638. 10.1007/s00415-021-10397-w 33449202 PMC7809551

[R67] Ona, E. D., Cano-de la Cuerda, R., Sanchez-Herrera, P., Balaguer, C., & Jardon, A. (2018). A review of robotics in neurorehabilitation: Towards an automated process for upper limb. Journal of Healthcare Engineering, 2018, 9758939. 10.1155/2018/9758939 29707189 PMC5901488

[R68] Padfield, N., Camilleri, K., Camilleri, T., Fabri, S., & Bugeja, M. (2022). A comprehensive review of endogenous EEG-based BCIs for dynamic device control. Sensors, 22(15), 5802. 10.3390/s22155802 35957360 PMC9370865

[R69] Paloschi, D., Bravi, M., Schena, E., Miccinilli, S., Morrone, M., Sterzi, S., Saccomandi, P., & Massaroni, C. (2021). Validation and assessment of a posture measurement system with magneto-inertial measurement units. Sensors, 21(19), 6610. 10.3390/s21196610 34640930 PMC8513009

[R70] Park, E., Yun, B. J., Min, Y. S., Lee, Y. S., Moon, S. J., Huh, J. W., Cha, H., Chang, Y., & Jung, T. D. (2019). Effects of a mixed reality-based cognitive training system compared to a conventional computer-assisted cognitive training system on mild cognitive impairment: A pilot study. Cognitive and Behavioral Neurology, 32(3), 172–178. 10.1097/wnn.0000000000000197 31517700

[R71] Pau, M., Caggiari, S., Mura, A., Corona, F., Leban, B., Coghe, G., Lorefice, L., Marrosu, M. G., & Cocco, E. (2016). Clinical assessment of gait in individuals with multiple sclerosis using wearable inertial sensors: Comparison with patient-based measure. Multiple Sclerosis and Related Disorders, 10, 187–191. 10.1016/j.msard.2016.10.007 27919488

[R72] Pearson, O. R., Busse, M., Van Deursen, R. W. M., & Wiles, C. M. (2004). Quantification of walking mobility in neurological disorders. QJM: An International Journal of Medicine, 97(8), 463–475. 10.1093/qjmed/hch084 15256604

[R73] Peretti, A., Amenta, F., Tayebati, S. K., Nittari, G., & Mahdi, S. S. (2017). Telerehabilitation: Review of the state-of-the-art and areas of application. JMIR Rehabilitation and Assistive Technologies, 4(2), e7. 10.2196/rehab.7511 28733271 PMC5544892

[R74] Perez-Marcos, D., Bieler-Aeschlimann, M., & Serino, A. (2018). Virtual reality as a vehicle to empower motor-cognitive neurorehabilitation. Frontiers in Psychology, 9. 10.3389/fpsyg.2018.02120 PMC6224455 30450069

[R75] Phu, S., Vogrin, S., Al Saedi, A., & Duque, G. (2019). Balance training using virtual reality improves balance and physical performance in older adults at high risk of falls. Clinical Interventions in Aging, 14, 1567–1577. 10.2147/cia.s220890 31695345 PMC6717859

[R76] Porter, M. E., & Heppelmann, J. E. (2017). Why every organization needs an augmented reality strategy. Harvard Business Review - HBR's 10 Must, 85(November-December 2017). https://hbr.org/2017/11/why-every-organization-needs-an-augmented-reality-strategy

[R77] Prasad, G., Herman, P., Coyle, D., McDonough, S., & Crosbie, J. (2010). Applying a brain-computer interface to support motor imagery practice in people with stroke for upper limb recovery: A feasibility study. Journal of NeuroEngineering and Rehabilitation, 7(1), 1–17. 10.1186/1743-0003-7-60 21156054 PMC3017056

[R78] Rau, C.-L., Chen, Y.-P., Lai, J.-S., Chen, S.-C., Kuo, T.-S., Jaw, F.-S., & Luh, J.-J. (2013). Low-cost tele-assessment system for home-based evaluation of reaching ability following stroke. Telemedicine and e-Health, 19(12), 973–978. 10.1089/tmj.2012.0300 24138613 PMC3850429

[R79] Rogante, M., Grigioni, M., Cordella, D., & Giacomozzi, C. (2010). Ten years of telerehabilitation: A literature overview of technologies and clinical applications. NeuroRehabilitation, 27(4), 287–304. 10.3233/NRE-2010-0612 21160118

[R80] Saladino, M. L., Gualtieri, C., Scaffa, M., Lopatin, M. F., Kohler, E., Bruna, P., Blaya, P., Testa, C., López, G., Reyna, M., Piedrabuena, R., Mercante, S., Barboza, A., & Cáceres, F. J. (2023). Neuro rehabilitation effectiveness based on virtual reality and tele rehabilitation in people with multiple sclerosis in Argentina: Reavitelem study. Multiple Sclerosis and Related Disorders, 70, 104499. 10.1016/j.msard.2023.104499 36645996

[R81] Sanchez-Vives, M. V., & Slater, M. (2005). From presence to consciousness through virtual reality. Nature Reviews Neuroscience, 6(4), 332–339. 10.1038/nrn1651 15803164

[R82] Semprini, M., Laffranchi, M., Sanguineti, V., Avanzino, L., De Icco, R., De Michieli, L., & Chiappalone, M. (2018). Technological approaches for neurorehabilitation: From robotic devices to brain stimulation and beyond. Frontiers in Neurology, 9. 10.3389/fneur.2018.00212 PMC5900382 29686644

[R83] Smuck, M., Odonkor, C. A., Wilt, J. K., Schmidt, N., & Swiernik, M. A. (2021). The emerging clinical role of wearables: Factors for successful implementation in healthcare. npj Digital Medicine, 4(1), 45. 10.1038/s41746-021-00418-3 33692479 PMC7946921

[R84] Sosnoff, J., Sandroff, B., Pula, J., Morrison, S., & Motl, R. (2012). Falls and physical activity in persons with multiple sclerosis. Multiple Sclerosis International, 2012. 10.1155/2012/315620 PMC3432547 22966459

[R85] Srivastava, A., Swaminathan, A., Chockalingam, M., Srinivasan, M. K., Surya, N., Ray, P., Hegde, P. S., Akkunje, P. S., Kamble, S., Chitnis, S., Kamalakannan, S., Ganvir, S., & Shah, U. (2021). Tele-neurorehabilitation during the COVID-19 pandemic: Implications for practice in low- and middle-income countries. Frontiers in Neurology, 12, 667925. 10.3389/fneur.2021.667925 34690907 PMC8529345

[R86] Stasolla, F., Lopez, A., Akbar, K., Vinci, L. A., & Cusano, M. (2023). Matching assistive technology, telerehabilitation, and virtual reality to promote cognitive rehabilitation and communication skills in neurological populations: A perspective proposal. Technologies, 11(2), 43. 10.3390/technologies11020043

[R87] Suppa, A., Kita, A., Leodori, G., Zampogna, A., Nicolini, E., Lorenzi, P., Rao, R., & Irrera, F. (2017). L-dopa and freezing of gait in Parkinson's disease: Objective assessment through a wearable wireless system. Frontiers in Neurology, 8, 406. 10.3389/fneur.2017.00406 28855889 PMC5557738

[R88] Tressoldi, P. E., Brembati, F., Donini, R., Iozzino, R., & Vio, C. (2012). Treatment of dyslexia in a regular orthography: Efficacy and efficiency (cost-effectiveness) comparison between home vs clinic-based treatments. Europe's Journal of Psychology, 8(3), 375–390. 10.5964/ejop.v8i3.442

[R89] Truijen, S., Abdullahi, A., Bijsterbosch, D., van Zoest, E., Conijn, M., Wang, Y., Struyf, N., & Saeys, W. (2022). Effect of home-based virtual reality training and telerehabilitation on balance in individuals with Parkinson disease, multiple sclerosis, and stroke: A systematic review and meta-analysis. Neurological Sciences, 43(5), 2995–3006. 10.1007/s10072-021-05855-2 35175439 PMC9023738

[R90] Tulsulkar, G., Mishra, N., Thalmann, N. M., Lim, H. E., Lee, M. P., & Cheng, S. K. (2021). Can a humanoid social robot stimulate the interactivity of cognitively impaired elderly? A thorough study based on computer vision methods. The Visual Computer, 37(12), 3019–3038. 10.1007/s00371-021-02242-y 34345091 PMC8323964

[R91] Ustinova, K. I., Perkins, J., Leonard, W. A., & Hausbeck, C. J. (2014). Virtual reality game-based therapy for treatment of postural and co-ordination abnormalities secondary to TBI: A pilot study. Brain Injury, 28(4), 486–495. 10.3109/02699052.2014.888593 24702281

[R92] Ventura, S., Brivio, E., Riva, G., & Baños, R. M. (2019). Immersive versus non-immersive experience: Exploring the feasibility of memory assessment through 360° technology. Frontiers in Psychology, 10, 2509. 10.3389/fpsyg.2019.02509 31798492 PMC6868024

[R93] Voinescu, A., Sui, J., & Stanton Fraser, D. (2021). Virtual reality in neurorehabilitation: An umbrella review of meta-analyses. Journal of Clinical Medicine, 10(7), 1478. 10.3390/jcm10071478 33918365 PMC8038192

[R94] Weiss, A., Herman, T., Giladi, N., & Hausdorff, J. M. (2015). New evidence for gait abnormalities among parkinson's disease patients who suffer from freezing of gait: Insights using a body-fixed sensor worn for 3 days. Journal of Neural Transmission, 122(3), 403–410. 10.1007/s00702-014-1279-y 25069586

